# Induction and differentiation of adipose-derived stem cells from human buccal fat pads into salivary gland cells

**DOI:** 10.1007/s13577-016-0132-z

**Published:** 2016-02-03

**Authors:** Miyuki Kawakami, Hiroshi Ishikawa, Akira Tanaka, Izumi Mataga

**Affiliations:** Department of Oral and Maxillofacial Surgery, School of Life Dentistry at Niigata, The Nippon Dental University, 1-8 Hamaura-cho, Chuo-ku, Niigata, 951-8580 Japan; Department of NDU Life Sciences, School of Life Dentistry, The Nippon Dental University, 1-9-20 Fujimi, Chiyoda-ku, Tokyo, 102-0071 Japan; Department of Oral and Maxillofacial Surgery, Niigata Hospital, The Nippon Dental University, 1-8 Hamaura-cho, Chuo-ku, Niigata, 951-8580 Japan; Division of Cell Regeneration and Transplantation, Advanced Research Center, School of Life Dentistry at Niigata, The Nippon Dental University, 1-8 Hamaura-cho, Chuo-ku, Niigata, 951-8580 Japan

**Keywords:** Salivary glands, Regenerative, Buccal fat pads, ASCs, Fibroblasts

## Abstract

Atrophy or hypofunction of the salivary gland because of aging or disease leads to hyposalivation that affects patient quality of life by causing dry mouth, deterioration of mastication/deglutition, and poor oral hygiene status. Current therapy for atrophy or hypofunction of the salivary gland in clinical practice focuses on symptom relief using drugs and artificial saliva; therefore, there is still a need to develop new therapies. To investigate potential novel therapeutic targets, we induced the differentiation of salivary gland cells by co-culturing human adipose-derived stem cells isolated from buccal fat pads (hBFP-ASCs) with human salivary-gland-derived fibroblasts (hSG-fibros). We examined their potential for transplantation and tissue neogenesis. Following the culture of hBFP-ASCs and hSG-fibros, differentiated cells were transplanted into the submandibular glands of SCID mice, and their degree of differentiation in tissues was determined. We also examined their potential for functional tissue reconstitution using a three-dimensional (3D) culture system. Co-cultured cells expressed salivary-glandrelated markers and generated new tissues following transplantation in vivo. Moreover, cell reconstituted glandular structures in the 3D culture system. In conclusion, coculture of hSG-fibros with hBFP-ASCs led to successful differentiation into salivary gland cells that could be transplanted to generate new tissues.

## Introduction

Atrophy or hypofunction of the salivary gland can occur following radiotherapy for head and neck cancer, by obstructive defects in the salivary ducts, in chronic graft-versus-host disease after bone marrow transplant, or by age-related changes. Under these conditions, the salivary gland is markedly impaired, especially acinar cells, and atrophied or decreased cell numbers causes a loss of functional parenchymal tissue. This subsequently causes decreased saliva secretion (dry mouth) which significantly affects the quality of life due to deterioration of mastication/deglutition disorder and poor oral hygiene [1–3]. Current therapy for dry mouth caused by atrophy of the salivary gland in clinical practice is only symptomatic and includes transiently increasing the secretory capacity of residual acinar cells by drugs, or moisturizing dry mouth tissues with artificial saliva. Minimally invasive, radical therapy to improve quality of life has yet to be established [3–5].

Here, we examined salivary gland cell differentiation from stem cells using biomaterials based on a previous study [6] to develop a less-invasive therapy for dry mouth by regenerating salivary gland cells. Previously, mouse-derived early ES-6 (mEES-6) cells were differentiated by co-culture with human salivary-gland-derived fibroblasts (hSG-fibros) to express salivary gland markers [6, 7]. When these cells were transplanted into normal submandibular glands in SCID mice, near-normal salivary gland tissues were formed, indicating this method induced cell differentiation with biomaterials alone. Here, we investigated the applicability of this basic experimental approach to human tissue stem cells for application in humans.

The oral cavity contains a mass of specialized fatty tissue, the buccal fat pad (BFP), distinct from subcutaneous fat [8]. Human adipose-derived stem cells from BFP (hBFP-ASCs) were used as the cell source. The easy accessibility and rich vascularization of BFP is attractive for grafting, and is used widely in oral surgery to repair bone and periodontal defects [9–12]. Harvesting of BFP is a simple procedure requiring minimal incision and local anesthesia. Salivary gland fibroblasts were used for induction, even though the degree of atrophy varied among the collected salivary gland tissues, because their organ-specific characteristics might induce organ-derived cells. We investigated the induction and differentiation of salivary gland cells using a co-culture system with hBFP-ASCs and hSG-fibros to determine their use in cell engraftment.

## Materials and methods

### Tissue preparation and cell isolation/culture

This study was approved by the Ethics Committee of the Nippon Dental University, School of Life Dentistry at Niigata, Japan. Informed consent was obtained preoperatively from patients undergoing surgery for salivary gland cancer, and the minimum necessary amount of tissue was extracted during surgery. This study adhered to the amended Declaration of Helsinki. BFP and salivary gland (submandibular glands) tissues were extracted from four patients aged 48–54 years.

BFPs were washed in Hanks’ solution (Nissui, Tokyo, Japan) and minced with a scalpel (Crossfield, Japan). Cells were dissociated at 37 °C for 30 min in 0.01 % collagenase (Sigma-Aldrich, St. Louis, MO, USA)/0.05 % dispase (Life Technologies, Carlsbad, CA, USA), centrifuged, and the stromal vascular fraction [13] was cultured. To isolate undifferentiated cells, CD31^+^ (vascular component marker) cells (Miltenyi Biotec, Tokyo, Japan) were depleted by magnetic-activated cell sorting (Miltenyi Biotec). CD31^−^ cells were further isolated from the stromal vascular fraction of BFPs, cultured, and used as hBFP-ASCs. Cell identification was performed by cell surface marker analysis [14] and pluripotency evaluation [11].

Normal salivary gland tissues were washed with Hanks’ solution, sliced with a razor-type scalpel, and strips were cultured under static conditions. Outgrowing fibroblasts were separated by colonial cloning using the filter paper (Toyo Roshi Kaisha, Japan) method [15]. These cells were considered hSG-fibros, and after identification were used for immunostaining and RT-PCR. These cells were cultured in growth medium (GM) [Dulbecco’s modified Eagle’s medium (DMEM)/F12 (Life Technologies) supplemented with 15 % fetal bovine serum (FBS; Life Technologies), 10 µM non-essential amino acid solution (Life Technologies), 100 U/ml penicillin, 50 µg/ml streptomycin (Life Technologies), and 0.25 µg/ml amphotericin B (Fungizone; Life Technologies)] in a CO_2_ incubator (4.7 % CO_2_ + 95.3 % air). GM was changed twice weekly. During culture, cells were observed by inverted-phase contrast microscope (Olympus, Tokyo, Japan). For primary culture and subculture, 0.2 % trypsin–0.02 % EDTA/PBS(–) solution (Trypsin 250; Difco, Detroit, MI, USA) and Hanks’ solution were used. Cells were cultured in 60-mm dishes (Falcon Plastics, Franklin Lakes, NJ, USA). Manipulation of cellular aggregates in culture (primary culture, subculture, and cryopreservation) was accomplished using 5-ml disposable pipettes (Nippon Genetics, Tokyo, Japan) and 15-ml (Greiner-bio-one, Tokyo, Japan) and/or 50-ml centrifuge tubes (Falcon Plastics).

### Cell surface analysis

Cell-surface analysis of hBFP-ASCs was by flow cytometry [14]. Fluorescein isothiocyanate (FITC)-conjugated mouse monoclonal antibodies against CD14, CD90 and CD105 (Abcam, Cambridge, MA, USA) and CD34 and CD44 (Becton & Dickinson, San Jose, CA, USA) were used. Phycoerythrin-conjugated mouse IgG1 (Becton & Dickinson) was used as a negative control. Data acquisition and analyses were performed with Guava Express Plus (version 5.3) software (EMD Millipore Corporation, Billerica, MA, USA).

### Multilineage cell differentiation

hBFP-ASCs were induced to differentiate in osteogenic, adipogenic or chondrogenic medium for several weeks. To induce multilineage differentiation, hBFP-ASCs were seeded at 1 × 10^5^ cells per well in 6-well plates and maintained in GM until confluent. Induction and control media were changed twice weekly [11].

For osteogenic differentiation, hBFP-ASCs were cultured in DMEM, high-glucose (DMEM-hg; Life Technologies) supplemented with 15 % FBS, 10 nM dexamethasone (Sigma-Aldrich), 10 mM β-glycerophosphate (Wako Pure Chemical Industries, Osaka, Japan), and 100 µM ascorbate-2-phosphate (Wako Pure Chemical Industries). Osteogenesis was confirmed by staining with 1 % alizarin red S (Merck, Darmstadt, Germany) for calcium deposition.

For adipogenic differentiation, hBFP-ASCs were cultured in DMEM-hg supplemented with 15 % FBS, 0.5 mM isobutylmethylxanthine (Wako Pure Chemical Industries), 1 µM dexamethasone (Sigma-Aldrich), and 100 µM indomethacin (Sigma-Aldrich). Adipogenesis was confirmed by staining with Oil red-O (Sigma-Aldrich).

For chondrogenic differentiation, hBFP-ASCs were cultured in DMEM-hg supplemented with 1 % FBS, 6.25 µg/ml insulin (Wako Pure Chemical Industries), 10 ng/ml transforming growth factor-β1 (Peprotech, Rocky Hill, NJ, USA), and 50 nM ascorbate-2-phosphate (Wako Pure Chemical Industries). Chondrogenesis was confirmed by staining with toluidine blue (Muto Pure Chemicals Co. Ltd., Tokyo, Japan). Control cultures were maintained in DMEM supplemented with 15 % FBS lacking induction supplements.

### Induction of differentiation into salivary gland cells by co-culture

Co-SG cells were induced by co-culture with hBFP-ASCs and hSG-fibros. Before co-culture, the growth potential of hSG-fibros was eliminated by mitomycin C (10 µg/ml; Kyowa Hakko Kirin, Tokyo, Japan) in GM [16, 17]. Then, hSG-fibros were washed using Hanks’ solution and co-cultured with hBFP-ASCs. Co-culture medium was changed twice weekly. No differentiation-inducing materials were used. After co-culturing for 1 week, cultured cell morphology changed, and cells were isolated by colonial cloning. Filter paper soaked with trypsin 250 solution was placed directly onto clusters cells, then removed from plastic dishes, shaken into a fresh plastic dish where cells were transferred to medium [15], and subculture was continued. Cultured cells after cloning (co-SG cells) were used within three passages of culture for analysis and experiments. Salivary amylase activity of differentiated cells was measured by amylase assay (Abcam).

### Co-SG cell transplantation in vivo

Co-SG cells were transplanted into normal submandibular glands of mice (Jcl scid/scid 8-weeks-old, female; CLEA Japan, Japan) to examine whether co-SG cells that differentiated by co-culture with human-derived cells could be engrafted in vivo, and whether the cells differentiated into salivary glands in vivo. Under anesthesia, co-SG cells (1 × 10^5^ cells/0.2 ml Hanks’ solution/mouse) were injected with a 23-G needle through an incision into submandibular glands. Mice were euthanized approximately 1 month after transplantation, and transplanted submandibular glands were harvested. Methods for animal husbandry and sacrifice conformed to the code of ethics for experimental animals of the Nippon Dental University School of Life Dentistry at Niigata, Japan (Approval No. 139).

### Reconstitution of salivary gland tissues

Tissue reconstitution was examined by three-dimensional (3D) culture system to determine whether salivary gland-like tissue structures formed in vitro. For tissue reconstitution, hBFP-ASCs and hSG-fibros were 3D co-cultured with a collagen sponge (type I collagen sponge; Stem Inc., Tokyo, Japan). For culture, hSG-fibros were injected into the collagen sponge soaked in GM. After culture under static conditions for 2 weeks, cells were colonized, and co-SG cells differentiated from co-culture of hBFP-ASCs with hSG-fibros were injected into a collagen sponge colonized with hSG-fibros. After culture under static conditions for 1 week, the sponge was transferred to continuous culture (6 ml/min) by circumfusion apparatus to maintain a constant culture environment for 3 weeks. Medium (200 ml/bottle) was changed every 3 days. Cultured tissues were examined histologically. Salivary amylase activity was measured in reconstructed tissues.

### Chromosome analysis

Direct chromosome preparation was performed in the exponential growth phase of co-SG cells at passage 3. Subconfluent cells were incubated with 100 nM colcemid (Sigma-Aldrich) for 4 h at 37 °C, then harvested and centrifuged. Cells were resuspended in 70 mM KCl (Wako Pure Chemical Industries) at 1 × 10^4^ cells/ml for 20 min at 37 °C, centrifuged, and fixed with freshly prepared methanol:acetic acid (3:1; Wako Pure Chemical Industries) solution for 5 min at room temperature. Next, cells were centrifuged, and the supernatant was discarded. The pellet was incubated overnight at 4 °C and then fixed in methanol:acetic acid solution. Cell suspension in fixative was dropped onto a wet, cold micro-glass slide and stained with Giemsa solution (Wako Pure Chemical Industries). Fifty mitotic figures were chosen randomly, analyzed and counted for distribution and karyotype.

### Histological examination

Thoracotomy was performed under general anesthesia to harvest tissues from mice. After perfusion with Hanks’ solution, perfusion fixation was achieved with 4 % paraformaldehyde. Submandibular glands were extirpated, embedded in fixing solution, dehydrated, and embedded in paraffin. Tissue sections were prepared at 4-µm thickness for hematoxylin and eosin (HE; Wako Pure Chemical Industries) staining, Periodic Acid Schiff’s (PAS, Wako Pure Chemical Industries) staining, and immunohistochemistry (after deparaffinization). After washing with PBS (Takara Bio, Japan), samples were activated in 0.1 % trypsin at 37 °C for 30 min and blocked with 1 % bovine serum albumin (Sigma-Aldrich) at room temperature for 30 min. Samples were incubated with the following primary antibodies at 4 °C overnight: monoclonal mouse anti-human mitochondria (1:1000, Sigma-Aldrich); polyclonal rabbit anti-human amylase (1:500, Sigma-Aldrich); and polyclonal rabbit anti-mouse aquaporin 5 (AQP-5, 1:1000, Abcam). After washing with PBS, samples were incubated with anti-mouse or anti-rabbit fluorescein-conjugated secondary antibodies (diluted 1:1000, Alexa Fluor; Life Technologies) at room temperature for 2 h. For nuclear staining and inclusion, Vectashield mounting medium with DAPI (Vector Laboratories, Burlingame, CA, USA) was used. For negative controls, primary antibody was omitted during immunostaining.

### Immunocytochemistry

hSG-fibros and co-SG cells during passage 3 were seeded in Laboratory-Tek II chamber slides (Nalge Nunc, Roskilde, Denmark), cultured in GM, and stained 3–4 days later. Cells were fixed in 100 % methanol (Wako Pure Chemical Industries) at −30 °C for 10 min, washed with PBS three times, and incubated in 1 % bovine serum albumin at room temperature for 30 min. Cultures were incubated with the following primary antibodies overnight at 4 °C: monoclonal mouse anti-human vimentin (1:1000, Sigma-Aldrich), monoclonal mouse anti-human mitochondria (1:1000, Sigma-Aldrich), polyclonal rabbit anti-human amylase (1:1000, Sigma-Aldrich), polyclonal rabbit anti-human basic fibroblast growth factor (bFGF, 1:250, Abcam), and polyclonal sheep anti-human nerve growth factor (NGF; 1:200, Abcam). After washing with PBS, cells were incubated with anti-mouse, anti-rabbit or anti-sheep fluorescein-conjugated secondary antibodies (Alexa Fluor; Life Technologies) diluted 1:1000 at room temperature for 30 min. For nuclear staining and inclusion, Vectashield mounting medium with DAPI (Vector Laboratories) was used. For negative controls, primary antibody was omitted during immunostaining.

### Reverse-transcriptase polymerase chain reaction (RT-PCR)

Reverse-transcriptase polymerase chain reaction was performed to confirm salivary gland marker gene expression in hSG-fibros, co-SG cells, and cultured tissues. Total RNA was extracted and purified from each cell type by RNeasy mini kit (Qiagen, Hilden, Germany). Using 1 µg of total RNA, cDNAs were synthesized using a high-capacity cDNA Reverse Transcription kit (Life Technologies). Platinum PCR SuperMix (Life Technologies) and a Veriti Thermal Cycler (Life Technologies) were used for PCR amplification according to the manufacturers’ instructions. PCR conditions were as follows: initial denaturing step at 95 °C for 2 min, 35 repeated cycles of denaturing at 95 °C for 30 s, primer annealing at 54–58 °C for 30 s, and extension at 72 °C for 1 min, followed by a final extension at 72 °C for 10 min. PCR products were separated by 2 % gel electrophoresis (Nippon Gene Co. Ltd., Japan) and were visualized with UV after ethidium bromide (Life Technologies) staining.

The following primers were used: amylase, sense 5′-GGG ATT TGG AGG GGT TCA GG-3′, antisense 5′-TTC TGT CAC CCG GCC ATT AC-3′ (NM_004038); AQP-5, sense 5′-CGG GCT TTC TTC TAC GTG G-3′, antisense 5′-GCT GGA AGG TCA GAA TCA GCT C-3′ (NM_001651); bFGF, sense 5′-AGA AGA GCG ACC CTC ACA TCA-3′, antisense 5′-CGG TTA GCA CAC ACT CCT TTG-3′ (NM_002006); NGF, sense 5′-GGC AGA CCC GCA ACA TTA CT-3′, antisense 5′-CAC CAC CGA CCT CGA AGT C-3′ (NM_002506); vimentin, sense 5′-GGG ACC TCT ACG AGG AGG AG-3′, antisense 5′-CGC ATT GTC AAC ATC CTG TC-3′ (NM_003380); collagen type I, sense 5′-CCA AAT CTG TCT CCC CAG AA-3′, antisense 5′-TCA AAA ACG AAG GGG AGA TG-3′ (NM_000088); and prostate stem cell antigen (PSCA), a marker of undifferentiated cells [18], sense 5′-TGC TGC TTG CCC TGT TGA T-3′, antisense 5′-CCT GTG AGT CAT CCA CGC A-3′ (NM_005672). The internal control was glyceraldehyde-3-phosphate dehydrogenase (GAPDH), sense 5′-CGT CTT CAC CAC CAT GGA GA-3′, and antisense 5′-CGG CCA TCA CGC CAC AGT TT-3′ (NM_002046).

## Results

### Analysis of cultured cells

**Fig. 1:**
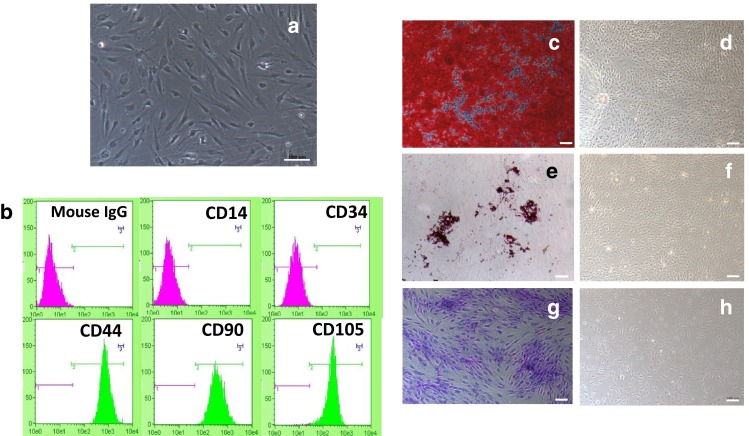
Identification of hBFP-ASCs. **a** Phase-contrast micrograph of spindle-shaped cells. *Scale bar* 100 µm. **b** Flow cytometric analysis of hBPP-ASCs with antibodies reactive to cell surface markers CD44, CD90, CD105, CD14 and CD34. Mouse IgG was used as an isotype control. **c**–**h** Differentiation of hBFP-ASCs to osteogenic, adipogenic and chondrogenic lineages. *Scale bars* 100 µm. **c** Alizarin red staining indicates mineralization after culture in osteogenic induction medium for 3 weeks. **d** No mineralized nodule formation was observed in control medium culture. **e** Oil red O staining indicates lipid clusters after culture in adipogenic induction medium for 4 weeks. **f** No lipid clusters were observed in control medium. **g** Toluidine blue indicates the induced clusters in chondrogenic induction medium for 4 weeks, **h** but no induction was seen in control cells

Cells isolated from tissues were identified before the experiment. Cells cultured and isolated from BFPs were observed under phase contrast microscopy and were spindle shaped (Fig. 1a). Specific cell markers of hBFP-ASCs characterized by flow cytometry (Fig. 1b) indicated they were strongly positive for CD44 (100 %), CD90 (99.8 %), and CD105 (94 %) (multipotent stromal cell markers), but negative for CD14 (0.1 %) and CD34 (0.03 %) (hematopoietic cell markers). To determine hBFP-ASC differentiation ability, they were subjected to osteogenic, adipogenic, and chondrogenic differentiation. After osteogenic induction, alizarin-red-positive mineralized nodules were observed in induced hBFP-ASCs (Fig. 1c), whereas no alizarin red staining was observed in controls (Fig. 1f). After adipogenic induction, clusters of Oil red O-positive cells were detected (Fig. 1d), but not in controls (Fig. 1g). After chondrogenic induction, clusters of toluidine-blue-positive cells were detected in induced cells (Fig. 1e), but not in controls (Fig. 1h).

**Fig. 2:**
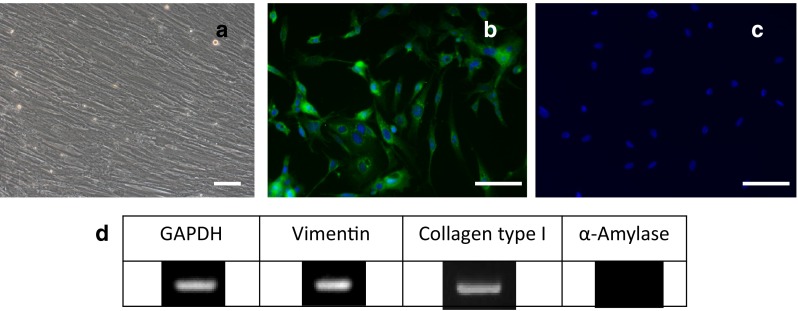
Identification of hSG-fibros. **a** Phase-contrast micrographs of typical spindle shapes. *Scale bar* 100 µm. **b**–**d** Immunostained images. DAPI (*blue*), **b** vimentin, and **c** amylase (*red*). **d** RT-PCR confirmed the cells were human-derived fibroblasts

Cultured fibroblasts with typical spindle-shaped morphology by phase-contrast microscopy (Fig. 2a) were analyzed by immunostaining (Fig. 2b) and RT-PCR (Fig. 2d), which indicated they were hSG-fibros. Amylase analysis by RT-PCR and immunostaining revealed no expression, indicating cells isolated from salivary glands did not include acinar cell components (Fig. 2c,d).

### Induction to salivary gland cells by co-culture (co-SG cells)

**Fig. 3:**
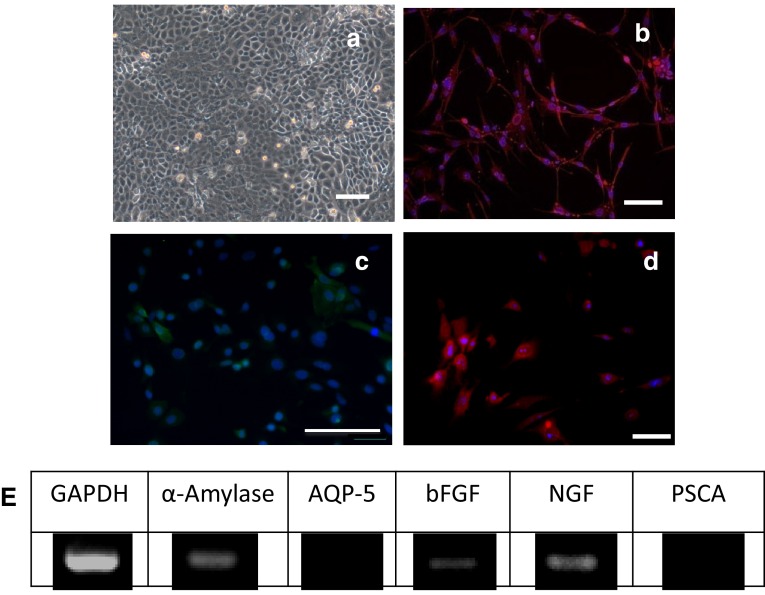
Identification of co-SG cells. **a** Phase-contrast micrographs of proliferating epithelial-like cells with tessellation. *Scale bar* 100 µm. **b**–**d** Immunostained images. DAPI (*blue*), **b** amylase (*red*), **c** bFGF (*green*), and **d** NGF (*red*) indicated salivary gland marker positivity. **e** RT-PCR showed expression of human-specific salivary gland markers

Cultured cell morphology was altered at 1 week after co-culture of hBFP-ASCs with hSG-fibros (Fig. 3a) compared with culture of hBFP-ASCs alone (Fig. 1a). Cells characterized by immunostaining (Fig. 3b–d) and RT-PCR (Fig. 3e) indicated salivary-gland-related markers (amylase, bFGF and NGF) were expressed indicating similar characteristics to salivary glands. AQP-5 expression was not seen by RT-PCR. Because PSCA expression was not observed, we concluded differentiation was induced in most cells, and undifferentiated cells were no longer present. In addition, amylase activity in salivary gland cells induced to differentiate was higher than in controls. We confirmed induced differentiation by functional assays (Fig. 7).

### Karyotype analysis of co-SG cells

**Fig. 4:**
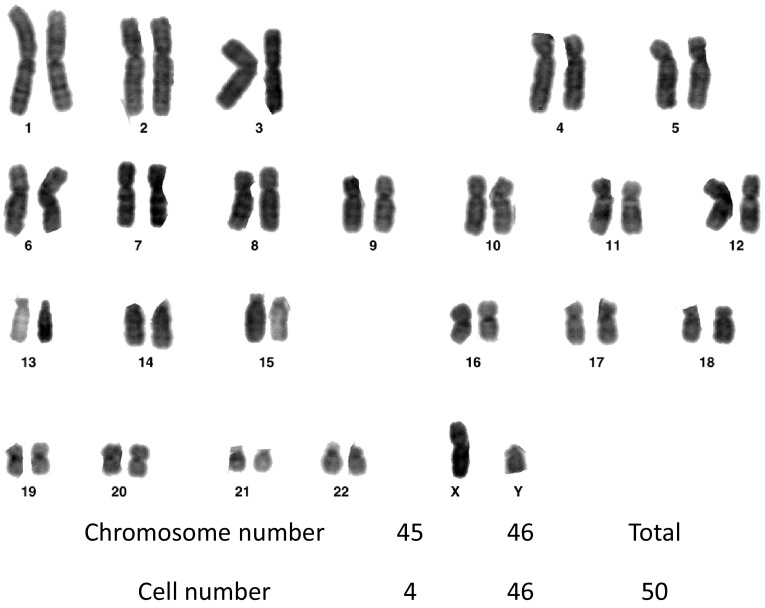
Karyotype analysis of co-SG cells. Maintenance of normal diploidy was confirmed by G-band analysis

To examine karyotype and chromosomal stability of cultured cells (passage 3), we performed G-banded karyotype analysis, which showed all samples had a normal (92 %) karyotype with diploid chromosome number (2*n* = 46; Fig. 4).

### Transplantation of co-SG cells in vivo

**Fig. 5:**
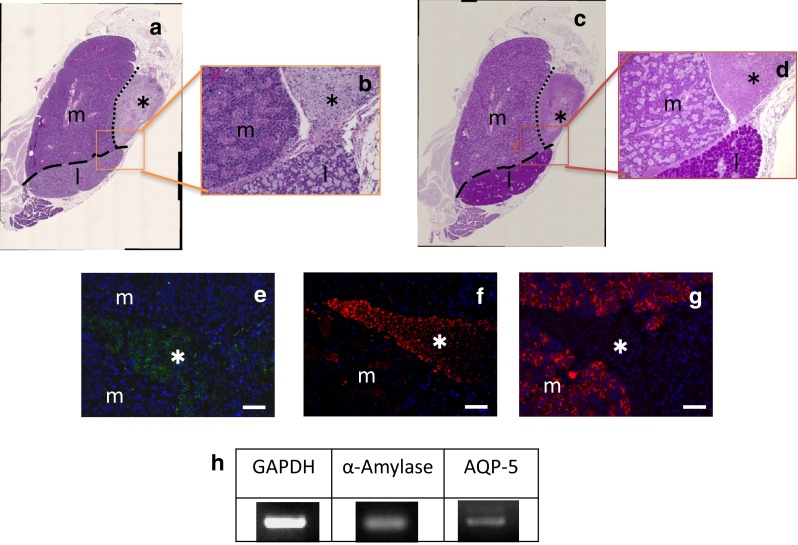
Transplantation of co-SG cells in vivo. **a**, **b** HE-stained images. **c**, **d** PAS-stained images. Regeneration of tissue (*asterisk*) in mice that differs in the vicinity of intrinsic glands within the same capsule. *Asterisk* regeneration of tissue, *m* submandibular gland, *l* sublingual gland. **e**–**g** Immunostained images. DAPI (*blue*), **e** human-specific mitochondria (*green*), **f** amylase (*red*), and **g** AQP-5 (*red*, mouse-specific antibody used) merged images of mitochondria and amylase, confirming the tissue was formed from human cells. *Asterisk* regeneration of tissue, *m* salivary gland of mouse. **h** RT-PCR showed expression of salivary gland markers. Primers for human-specific sequences were used

After confirming cells obtained from hBFP-ASCs and hSG-fibros co-culture were salivary gland cells, they were transplanted to normal submandibular glands of mice. About 1 month after transplantation, staining of the transplanted side of the submandibular gland with HE and PAS revealed tissue distinct from mouse salivary gland in the vicinity of the intrinsic glands within the same submandibular gland capsule (Fig. 5a–d, asterisk). Further analysis by immunostaining (asterisk) indicated a collection of cells positive for human-specific mitochondria and amylase and negative for mouse-specific AQP-5 (Fig. 5e–g). RT-PCR of transplanted tissue using human-specific primers also showed expression of amylase, a salivary gland marker (Fig. 5h). Moreover, expression of AQP-5, which was not seen in the co-SG cell state, was observed in tissues following transplantation. Thus, transplantation of co-SG cells induced by co-culture of hBFP-ASCs with hSG-fibros into normal tissues in vivo mediated regeneration of neo-salivary gland tissues that might produce amylase and have a functional role.

### Reconstitution of salivary gland tissues

**Fig. 6:**
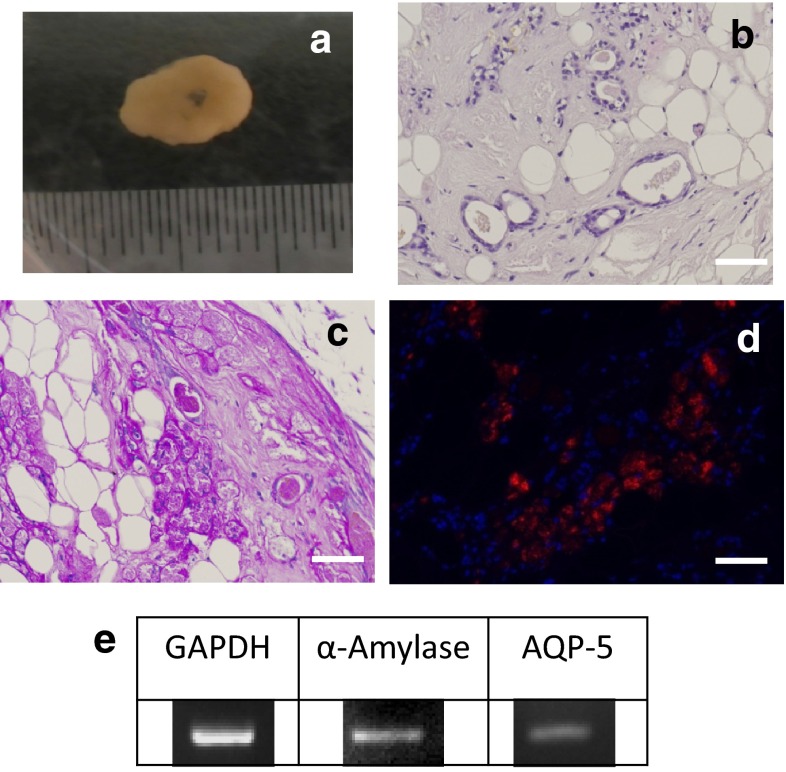
Reformation of salivary gland tissue in 3D cultures. **a** Macrophotograph. *Scale bar* 10 mm. **b** HE-stained cells. **c** PAS-stained cells. **d** Immunostained cells. DAPI (*blue*) and **d** amylase staining confirmed this was tissue formed from human-derived amylase-positive cells. **e** RT-PCR confirmed expression of salivary gland markers. Primers of human-specific sequences were used

**Fig. 7:**
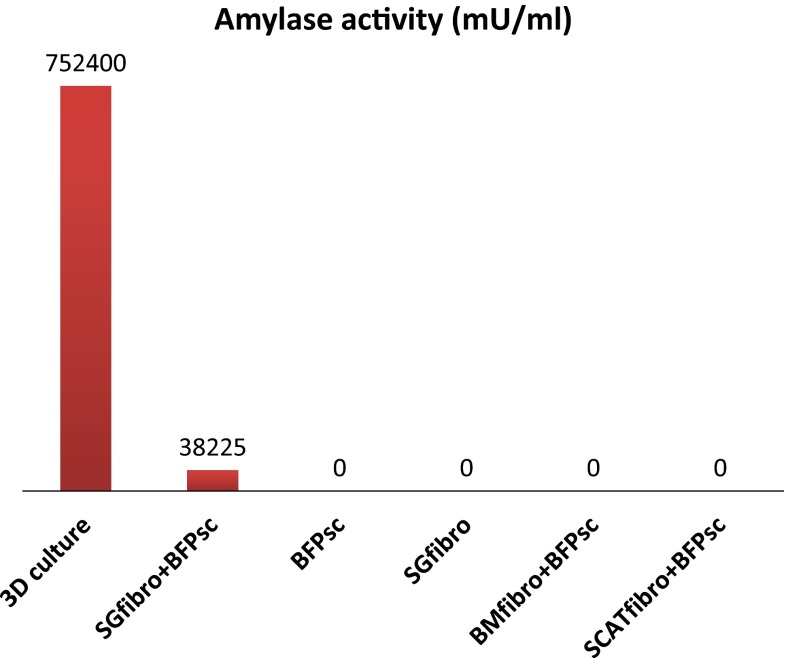
Amylase activity assay. *3D culture* reconstitution of salivary gland tissues, *SGfibro* *+* *BFPsc* co-cultured with hBFP-ASCs and hSG-fibro, *BFPsc* only hBFP-ASCs, *SGfibro* only SGfibro, *BMfibro* *+* *BFPsc* co-cultured with bone marrow-derived fibroblasts and hBFP-ASCs, *SCATfibro* *+* *BFPsc* co-cultured with subcutaneous fat-derived fibroblasts and hBFP-ASCs

Samples obtained from co-SG cells reconstituted by 3D culture examined by HE staining confirmed acinar-like or duct-like structures formed inside the sponge (Fig. 6a–c). PAS staining and immunostaining demonstrated the inside of the duct-like structure was amylase positive (Fig. 6d). Thus, co-SG cells induced by co-culture of hBFP-ASCs with hSG-fibros formed acinar-like or duct-like structures that produced amylase in a 3D culture. RT-PCR of these structures also showed expression of amylase and AQP-5 (salivary gland markers, Fig. 6e). Furthermore, amylase activity analysis confirmed activity in induced cells and 3D culture samples (Fig. 7).

## Discussion

There are currently no established radical therapies for atrophied and hypofunctioning salivary glands caused by age or illness. The main therapeutic options provide symptom relief (gargles, oral lubricants), or salivary stimulation using medication [3, 5]. These effects are not adequate and many patients suffer reduced quality of life associated with decreased saliva production [2, 3]. Recent studies have focused on regenerative medicine as a radical therapy for atrophy and hypofunction of salivary glands [1, 4, 19, 20]. Therefore, the transplant of salivary gland cells differentiated from stem cells in culture to regenerate salivary gland tissue, particularly solid organs containing acinar and duct systems, might be a future therapy for atrophied and hypofunctioning salivary glands. Clinically, promoting new growth and replacing salivary glands with cell transplants might be less invasive and more feasible than transplantation of glandular tissue (organs) formed in 3D cultures.

The most commonly reported method in salivary gland regenerative medicine is tissue stem cell transplantation [4, 19, 21, 22]. Ductal cells positive for several stem cell markers in the ductal compartment of salivary glands [4, 23, 24] were transplanted into salivary glands of a mouse model of radiation exposure to regenerate acinar cells and restore saliva volume [22, 25]. However, it might be difficult to obtain sufficient cell numbers because stem cells must be derived from salivary glands that might already be atrophied. Therefore, we investigated a method of isolating and transplanting glandular epithelial cells derived from normal salivary glands, although transplantation experiments showed that cells in salivary gland tissue similar to normal cells may not be successfully engrafted [26]. Preparation of transplantable cells with a high regenerative capacity is therefore desired. However, for isolating stem cells, individual culture conditions must be established depending on the condition of the extracted tissue. No simple, clinically applicable method is thought to exist.

Bone-marrow-derived stem cells were recently shown to differentiate into epithelial cells in vitro [18, 21, 27–29]. When stem cells isolated from salivary glands were transplanted in vivo into damaged and atrophied salivary glands, they remained in tissues and had salivary gland function [21, 30, 31]. However, issues remain with the establishment of a simple method for regenerative therapy. For example, even if adequate numbers of stem cells are transplanted, the number of cells engrafted is limited owing to inhibition of cell adhesion [26, 32], and tissue regeneration does not occur unless a certain level of function remains before transplantation. In addition, bone marrow extraction is difficult.

To produce transplantable cells with high regenerative capacity, we investigated a method to induce differentiation into salivary gland cells by co-culturing hBFP-ASCs with hSG-fibros. These differentiated cells from hBFP-ASCs co-culture were transplanted into normal mouse submandibular glands to form new salivary gland tissues. We showed that stem cells with pluripotent potential isolated from BFPs extracted during dentistry are a viable source of transplantable cells for regenerative medicine. Thus, salivary gland cells induced to differentiate by the co-culture system can be grafted and regenerate tissue without inducing cell inhibitory mechanisms. Furthermore, this method has the following advantages: (1) stable phenotype; and (2) induction, differentiation and transplantation can be repeated because cells can be cryopreserved.

The amylase assay kit is intended for quantifying amylase metabolites. Thus, it is possible to induce the differentiation of salivary gland cells that secrete amylase with physiological activity.

Regarding reconstruction of tissues in 3D culture, salivary gland cells induced to differentiate by the co-culture method were confirmed in vitro to produce salivary-gland-like tissues. A high tissue regenerative capacity was also seen in vivo. Improving culture methods will enhance this potential source of transplantable tissues for salivary glands to repair partial defects from surgery, if structure precision can be increased. Thus, we are planning to investigate whether the stability of salivary gland tissue formed in 3D culture can be maintained in vivo.

For clinical applications, BFP and salivary gland tissues should be extracted with minimal invasiveness from patients with atrophied and hypofunctioning salivary glands, to isolate stem cells and fibroblasts from each tissue, induce their differentiation into salivary gland cells by co-culture, and return the cells to the original patient. For greater safety, we regeneration of hypofunctioning salivary glands might be assisted by administering conditioned medium from salivary gland cells induced to differentiate by co-culture. We aim to develop a treatment using conditioned medium without cell components.

In conclusion, we successfully induced differentiation of cells to salivary gland-type cells by co-culturing hBFP-ASCs with hSG-fibros. This method is minimally invasive and safe for tailor-made salivary gland regeneration therapy.
